# Analysis of perovskite oxide etching using argon inductively coupled plasmas for photonics applications

**DOI:** 10.1186/s11671-021-03494-2

**Published:** 2021-02-10

**Authors:** Guanyu Chen, Eric Jun Hao Cheung, Yu Cao, Jisheng Pan, Aaron J. Danner

**Affiliations:** 1 Department of Electrical and Computer Engineering, National University of Singapore, 4 Engineering Drive 3, Singapore, 117583 Singapore; 2 Institute of Materials Research and Engineering, A∗STAR (Agency for Science, Technology and Research), 2 Fusionopolis Way, Innovis #08-03, Singapore, 138634 Singapore

**Keywords:** Perovskite oxide, Argon, Inductively coupled plasma etching, Photonics

## Abstract

We analyzed the dry etching of perovskite oxides using argon-based inductively coupled plasmas (ICP) for photonics applications. Various chamber conditions and their effects on etching rates have been demonstrated based on Z-cut lithium niobate (LN). The measured results are predictable and repeatable and can be applied to other perovskite oxides, such as X-cut LN and barium titanium oxide (BTO). The surface roughness is better for both etched LN and BTO compared with their as-deposited counterparts as confirmed by atomic force microscopy (AFM). Both the energy-dispersive X-ray spectroscopy (EDS) and X-ray photoelectron spectroscopy (XPS) methods have been used for surface chemical component comparisons, qualitative and quantitative, and no obvious surface state changes are observed according to the measured results. An optical waveguide fabricated with the optimized argon-based ICP etching was measured to have -3.7 dB/cm loss near 1550 nm wavelength for Z-cut LN, which validates this kind of method for perovskite oxides etching in photonics applications.

## Introduction

Silicon photonics has experienced great development in recent decades due to its low cost and large scale integrability [1]. However, the lack of a Pockels effect restricts some of its applications due to its centrosymmetric crystal structure [2]. Perovskite oxides such as LiNbO_3_ (LN) and BaTiO_3_ (BTO) have thus received great attention for photonics applications due to their large Pockels effects [3–10]. Various LN- and BTO-based photonic devices have been demonstrated with superior performance [3–10]. For such application, a waveguide having a high refractive index contrast is the basic component for light confinement [11]. Traditionally, waveguides have been formed in LN through ion diffusion [12], which has allowed only a low refractive index contrast and poor resulting optical confinement. The breakthrough of LN-based integrated devices relies on direct dry etching of LN thin films [3, 6–10]. However, there is no comprehensive analysis of LN dry etch methods reported up to now. On the other hand, BTO has an even higher Pockels coefficient of 1640 pm/V than LN (~ 30 pm/V) [2], which can support much better photonic devices. However, directly etched BTO-based photonics devices have not yet been demonstrated, which is probably due to it being difficult to etch. The most common method of creating higher-index waveguides in these materials in literature is to first deposit a layer of easy-to-etch material, then transfer the pattern onto this layer through dry etching. The resulting optical mode only partially overlaps with the lower unetched BTO layer, thus degrading its performance; such a method cannot maximize its Pockels effect [4, 5]. Although fluorine and chloride-based plasma etching of BTO has been proposed for semiconductor memory applications [13–15], reaction products which can be redeposited on the surfaces and sidewall during the etching process reduces suitability of such etching chemistries for photonics applications.

Therefore, argon plasma-based inductively coupled plasma (ICP) etching of perovskite oxides LN and BTO is comprehensively analyzed in this manuscript. Different factors and their effect on the etching rates are compared for both LN and BTO. The surface roughness before and after etching is analyzed through atom force microscopy (AFM). Energy-dispersive X-Ray spectroscopy (EDS) and X-ray photoelectron spectroscopy (XPS) methods are used for characterizing surface chemical state changes. It is observed that the surface is much smoother with no etching residue detected in both etched LN and BTO samples. Based on optimized etching conditions, optical waveguides are etched on a Z-cut LN sample, with a measured -3.7 dB/cm loss, which validates the argon-based ICP method in its suitability for general perovskite oxides etching in photonics applications.

## Experiment and results

### Methods

**Fig. 1:**
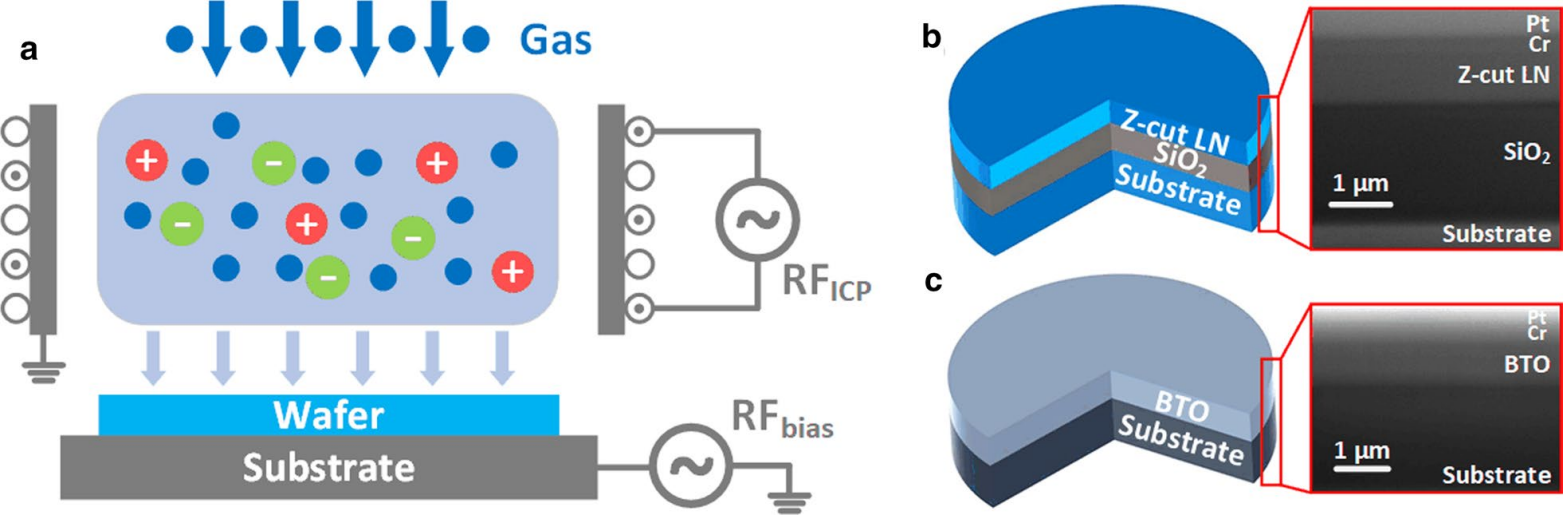
**a** Schematic structure of ICP process. The epitaxy structures and SEM images of **b** Z-cut LN and **c** BTO used in the experiment (Pt and Cr are deposited as protective layers for FIB cutting)

A 13.56 MHz Oxford PlasmaPro 100c Cobra is used for the argon-based ICP etch experiment, and a schematic structure of the etching is shown in Fig. 1a. The input gas is ionized under time-varying electromagnetic fields, which are produced by an inductively coupled coil under the first radio frequency source (RF_ICP_). The generated plasma ions accelerate vertically toward the bottom wafer under bias voltage, which is controlled by the second RF source (RF_bias_) connected to the substrate holder/electrode. The volatile etching gas products are discharged thorough a vent. Z-cut LN is used as an example for demonstration of the relationship between different process conditions and etch rate; the LN epitaxial structure is shown in Fig. 1b. The thickness of the top lithium niobate layer and silicon oxide are 700 nm and 2 µm, respectively. A 50 nm chrome (Cr) layer is first deposited by electron beam (e-beam) evaporation onto the sample for the facilitation of lithography. Then, about 1 µm of ma-N 1400 photoresist is spin-coated on the surface by photolithography using a laser writer. After development and subsequent Cr wet etch, the patterned structure serves as a shadow mask for dry etching. After ICP etching, this mask is stripped in hot N-methyl-2-pyrrolidone and Cr etchant. Feature depths before etching, after etching and after shadow mask removal are recorded through a surface profiler at fixed points, and the etch rates of the photoresist and Z-cut LN are calculated accordingly.

### Etch rates analysis

**Fig. 2:**
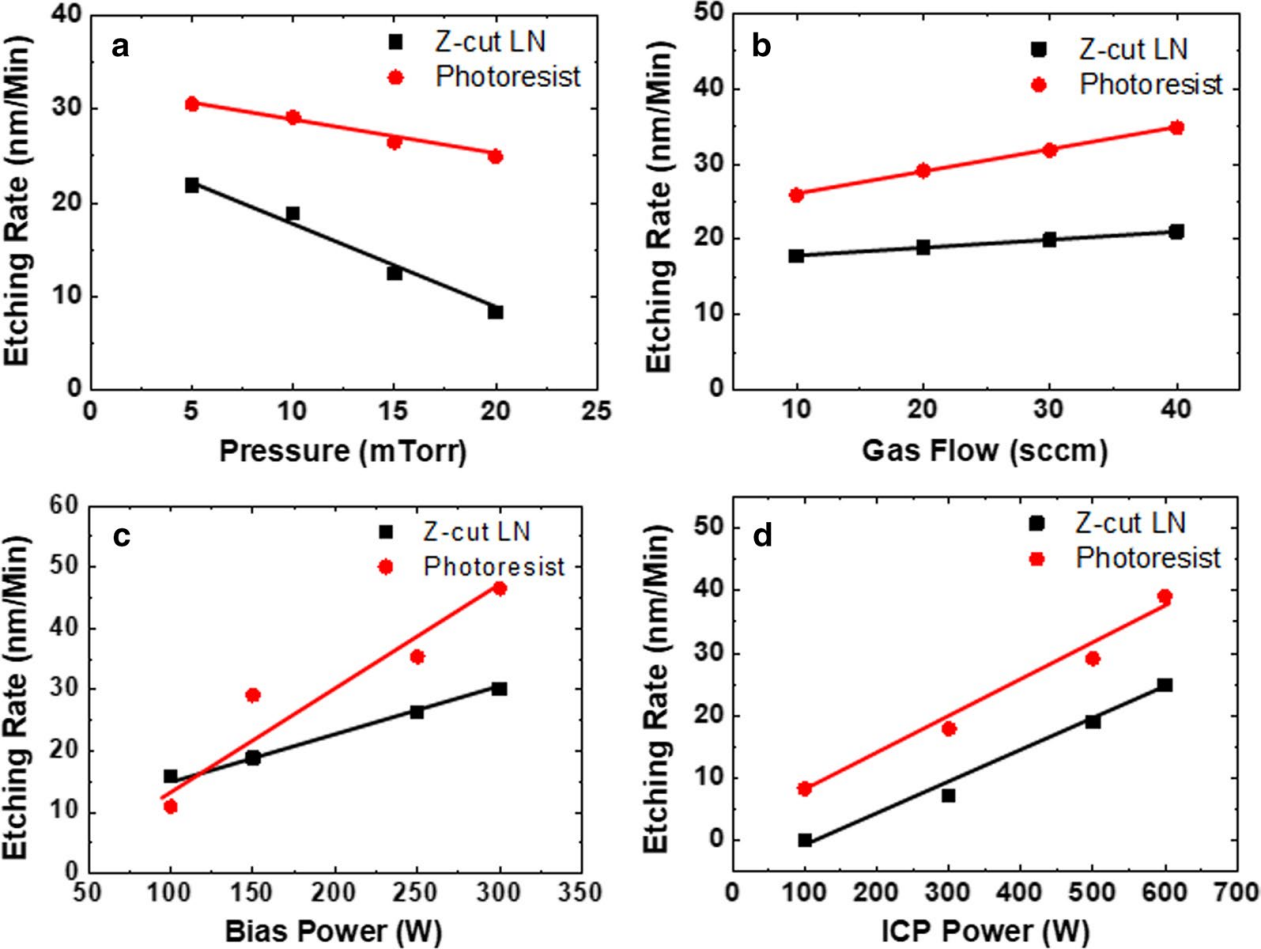
Etch rates of Z-cut LN and photoresist under different **a** pressure, **b** gas flow, **c** bias power and **d** ICP power

Four factors are validated during the experiment: chamber pressure, gas flow rate, bias power and ICP power. Before each etch, a 5 min pure oxygen plasma treatment is performed for chamber cleaning. Only argon gas is used during the etching, and the base etching condition is: 5 mTorr chamber pressure, 20 sccm gas flow, 150 W bias power and 500 W ICP power. The measured etch rates of Z-cut LN and photoresist with respect to different combined conditions are shown in Fig. 2. It can be observed from Fig. 2a that the etch rate of Z-cut LN increases when the chamber pressure is reduced, and the increase in the Z-cut LN etch rate is relatively linear (the slope is about 0.95 nm/min per mTorr pressure decrease) and predictable. It should be noted that the surface profile depth measurement of Z-cut LN is more accurate than with the photoresist, because the surface photoresist after etching is not as flat as the Z-cut LN. When the chamber pressure is lower, the random collision motion is reduced and the argon ions are transported more orderly, which can explain why higher etch rates are observed under lower chamber pressure. The etch rate increases linearly for Z-cut LN with respect to gas flow conditions, as shown in Fig. 2b, which means more argon plasma is activated when the gas flow is larger. The slope of the Z-cut LN etch rate is about 0.11 nm/min per unit sccm gas flow increase, as can be concluded from Fig. 2b. Both the etch rate of Z-cut LN and photoresist increase when the bias and ICP power increase, as can be seen from Fig. 2c, d. When the ICP power is as low as 100 W, few argon atoms are ionized and the etch rate of Z-cut LN is quite small, as shown in Fig. 2d. More argon atoms will be ionized when the ICP power is increased, and thus a higher etching rates result. With an increase in bias, the ion acceleration speed will also be larger as the electric field is stronger. Both an increase in plasma density as well as ion acceleration will result in higher etching rates, which can be found in Fig. 2c, d. The slopes are about 0.072 and 0.059 nm/min per watt of bias and ICP powers, respectively. The nonlinear curve of photoresist etching rate with relation of bias power is probably due to the measurement error resulting from a surface that’s not flat.

In Fig. 2a, the 21.87 nm/min etch rate is obtained under the conditions of 5 mTorr chamber pressure, 20 sccm gas flow, 150 W bias power and 500 W ICP power. About 37 nm/min etch rate is measured with conditions of 10 mTorr chamber pressure, 30 sccm gas flow, 300 W bias power and 700 W ICP power, which is highly consistent with the calculated results (40.4 nm/s) based on measured data shown in Fig. 2. Therefore, it can be concluded that the etch rate of Z-cut LN is regular and predicable.

**Fig. 3:**
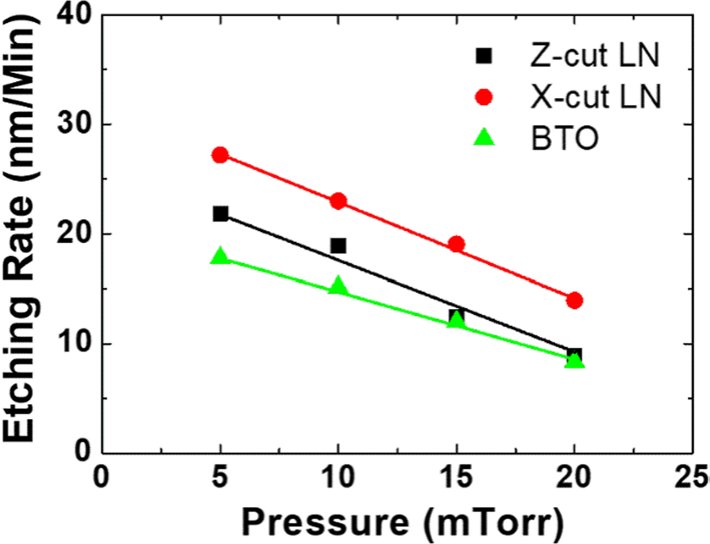
Comparison result of etch rates with respect to chamber pressure for BTO, Z-cut and X-cut LNs

Based on the same base conditions (5 mTorr chamber pressure, 20 sccm gas flow, 150 W bias power and 500 W ICP power), a similar etching experiment involving X-cut LN and BTO is carried out to examine the effect of the chamber pressure (as an example of different conditions), and the measured data is shown in Fig. 3. The X-cut LN used here is a bulk crystal, whereas the BTO is an epitaxial layer grown on a dysprosium scandate (DSO) substrate using pulsed laser deposition (PLD), with structure shown in Fig. 1c. When the chamber pressure is reduced, both the etch rate of BTO and X-cut LN increase, which accord well with the Z-cut LN results. The slightly different observed slope can be attributed to the small differences in crystal quality. It can thus be concluded that the etching parameters in Fig. 2 are also broadly suitable for perovskite oxide type X-cut LN and BTO.

### Surface morphology analysis

**Fig. 4:**
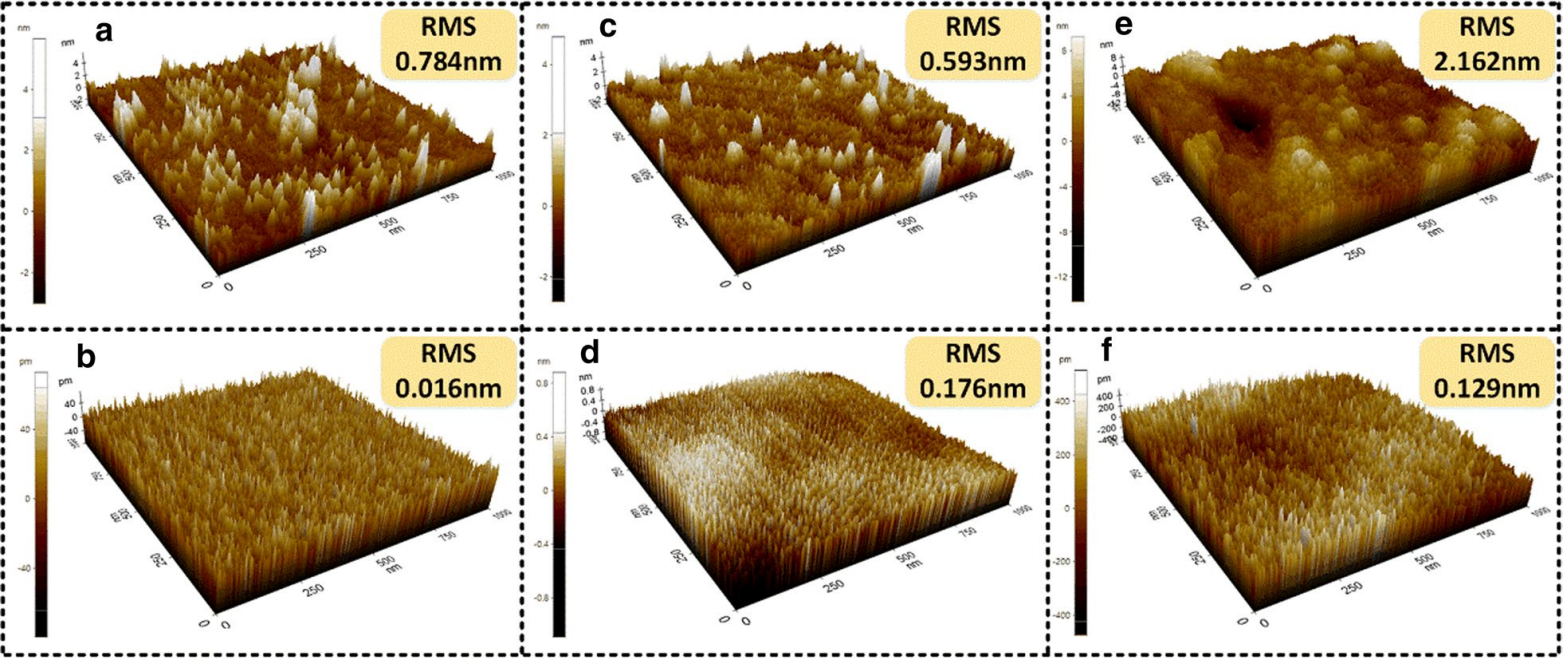
The 3D view of surface morphologies of Z-cut LN, X-cut LN and BTO measured by AFM. **a** As-deposited and **b** etched Z-cut LN. **c** As-deposited and **d** etched X-cut LN. **e** As-deposited and **f** etched BTO

In order to evaluate any potential surface morphology changes caused by etching, AFM is used here with the scan area set to $1\times 1 {\mathrm{\mu m}}^{2}$. AFM pictures of as-deposited and after-etching for Z-cut LN, X-cut LN and BTO are shown in Fig. 4. It can be found from Fig. 4a, b that the etched Z-cut LN has nearly one order lower surface root-mean-square (RMS) roughness compared with the as-deposited sample. For X-cut LN and BTO, same smoother surface after etching can be found from Fig. 4c–f. The slightly larger RMS roughness for as-deposited BTO is due to the original growth quality, as the LN sample is a commercial product and the BTO film is grown in our laboratory on a substrate (DSO) which itself may not have minimized surface roughness. The lower RMS roughness of the etched sample can be attributed to the physical etching property of argon plasma-based ICP, which makes the etch process a bit like grinding/polishing.

### Surface state analysis

**Fig. 5:**
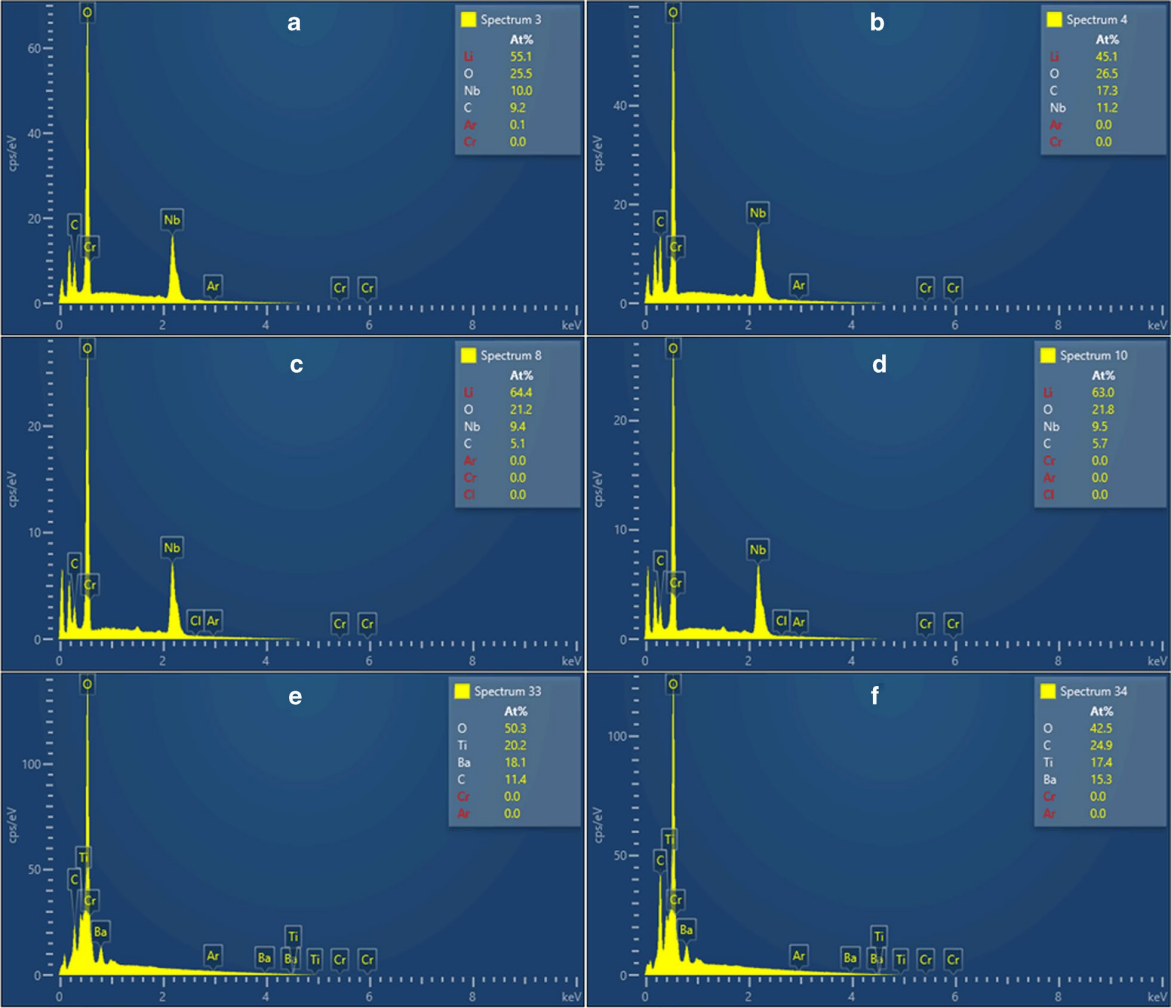
The measured EDS results. **a** As-deposited and **b** etched Z-cut LN samples; **c** as deposit and **d** etched X-cut LN samples; **e** as deposit and **f** etched BTO samples

EDS analysis is carried out for the as-deposited and etched Z-cut LN, X-cut LN and BTO samples, to qualitatively analyze any possible surface component changes, and the measured results are shown in Fig. 5. During the measurement, Lithium (Li), Niobate (Nb), Oxygen (O), Carbon (C), Argon (Ar) and Chrome (Cr) are recorded for Z-cut and X-cut LN, as shown in Fig. 5a–d, while for BTO sample, Barium (Ba), Titanium (Ti), O, C, Ar and Cr are analyzed, as shown in Fig. 5e, f. Compared with the as-deposited samples, no significant difference in elemental constituent is observed from Fig. 5. There is no residual argon within the etched area in any of the etched samples, which shows that argon plasma-based ICP is purely a physical process causing no unexpected secondary chemical changes, and no etching reactants are produced.

**Fig. 6:**
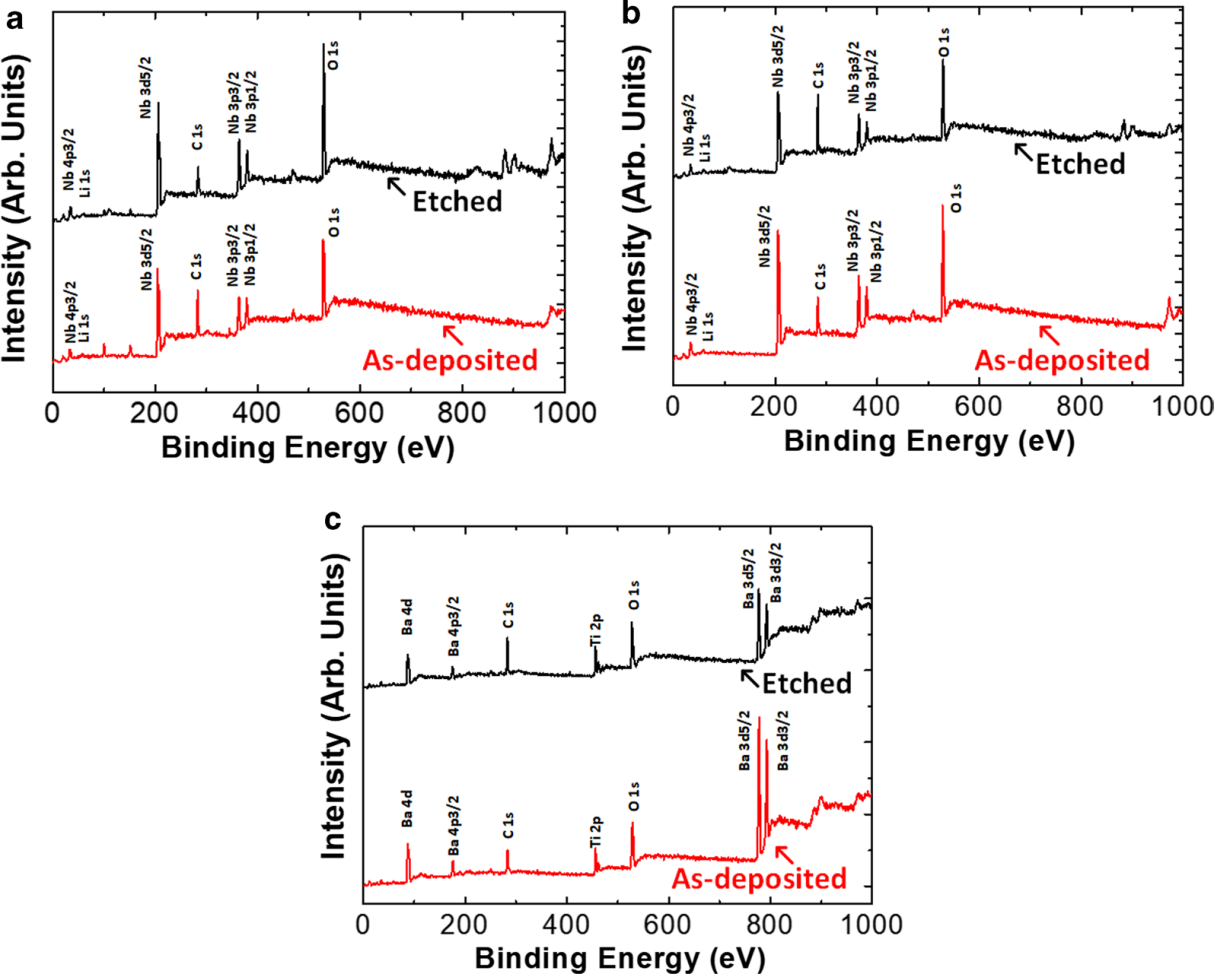
XPS survey spectra of **a** Z-cut LN, **b** X-cut LN and **c** BTO before and after etching. Bottom (red) and top (black) lines represent the as-deposited and etched samples

In order to further analysis of any surface chemical composition changes, XPS analysis was performed. Measurements were carried out in a Thermo Fisher Scientific Theta Probe system equipped with a monochromatic, micro-focused Al K $\alpha$ (1486.6 eV) X-ray source and XPS spectra were recorded at a detection angle (q) of 50, with respect to the sample surface. The base pressure of analysis chamber is $5\times {10}^{-10}$ mbar. Figure 6 shows the XPS survey spectra of Z-cut LN, X-cut LN and BTO samples range from 0 to 1000 eV binding energy (BE). There are Li 1 s, Nb 3p_1/2_, Nb 3p_3/2_, Nb 3d_5/2_, Nb 4p_3/2_, O 1 s, and C 1 s for both Z-cut LN and X-cut LN, as shown in Fig. 6a, b. In Fig. 6c, there are Ba 4d, Ba 4p_3/2_, C 1 s, Ti 2p, O 1 s, Ba 3d_5/2_ and Ba 3d_3/2_ for BTO. The XPS spectra reported here are referenced to the BE of C–C/C-H component peak of C 1 s spectra at 285.0 eV [13, 16]. It can be observed from Fig. 6 that there is no big difference in the survey spectra for as-deposited and etched samples. Some small peaks after etching are caused by the minor contamination during sample processing and storage.

**Fig. 7:**
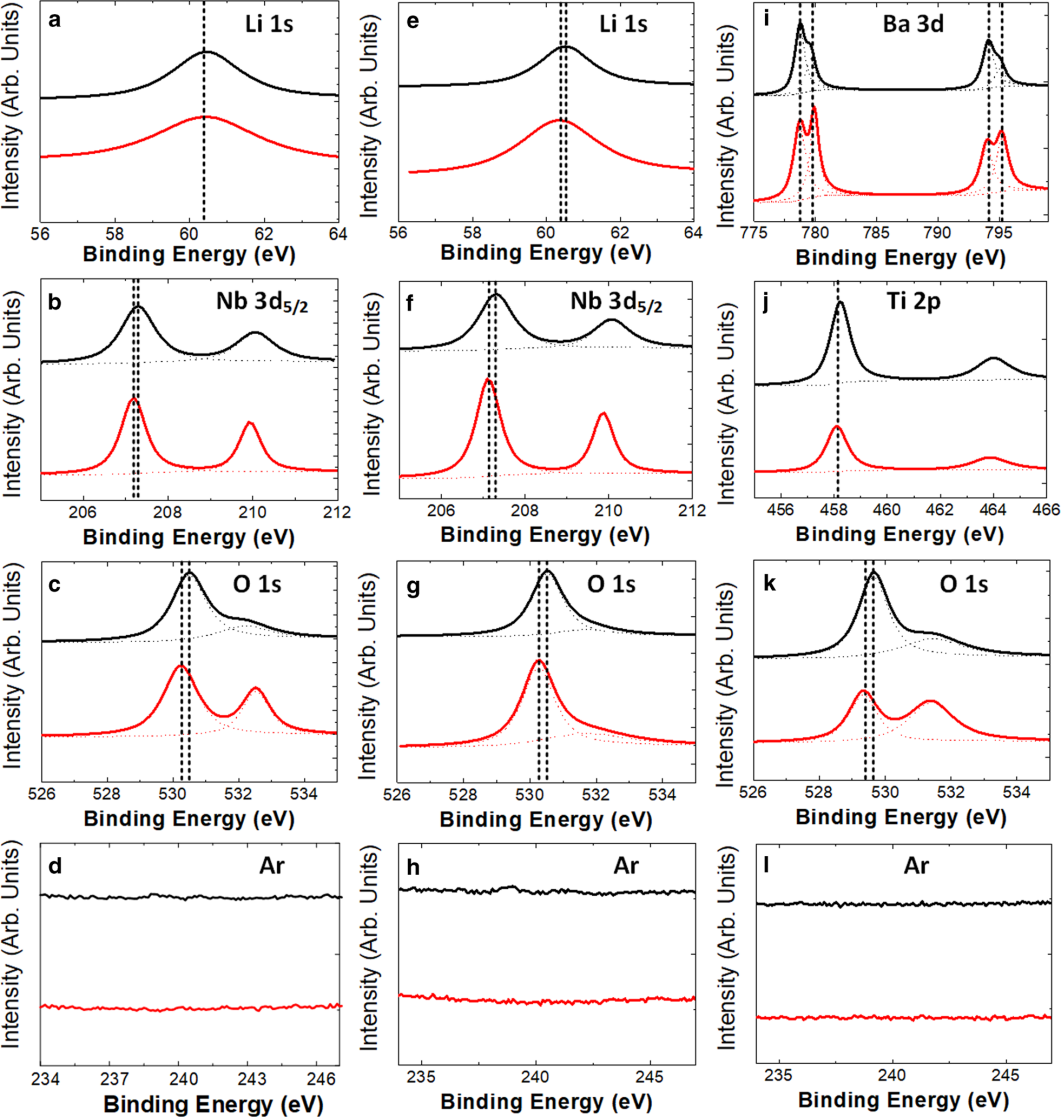
High resolution spectra for as-deposited and etched Z-cut LN, X-cut LN and BTO samples. **a** to **d** represent the Li, Nb, O, Ar for Z-cut LN. **e**–**h** represent the Li, Nb, O, Ar for X-cut LN. **i** to **l** represent the Ba, Ti, O, Ar for BTO. Bottom (red) and top (black) lines represent the as-deposited and etched results in every single picture

To further identify chemical state of each element in detail, the high-resolution spectra of all elements presented on surface were acquired and fitted after Shirley-type secondary electron background subtraction [13]. The fitting results are shown in Fig. 7. Figure 7a to d are Li 1 s, Nb 3d_5/2_, O 1 s and Ar for Z-cut LN. There are no obvious changes for the peak of Li 1 s, as shown in Fig. 7a. Compared with the as-deposited sample, the peaks of Nb 3d_5/2_ and O 1 s change, respectively, by 0.1 and 0.2 eV towards higher BEs in the etched case, as shown in Fig. 7b, c. Such small changes are close to the measurement error and indicate that there is no obvious chemical state change for Nb and O. It's noted that there are two component peaks for fitting of O 1 s spectrum, and the main peak around 530.2 eV is from Nb–O bonds. The other sub-peak around 532.5 eV can be attributed to contamination, as it is removed by the physical bump in the vacuum chamber and thus results weaker signal strength [13]. No obvious argon peak is observed in both the as-deposited and etched samples, which validates that the argon-based ICP etching resulted in no residue from etching.

Figures 7e–h show Li 1 s, Nb 3d_5/2_, O 1 s and Ar for X-cut LN. The conclusion is similar as in Z-cut LN. All peaks of Li 1 s, Nb 3d_5/2_ and O 1 s shift 0.2 eV towards higher BEs for the etched samples compared with as-deposited ones. For Ar 2p spectrum, there is one small peak in the etched sample around 239.0 eV, which can attribute to the residual argon. Although Ar will not chemically react with the etched material, the high energy ion will be implanted into the etched surface during etching process. And such an implantation is expected to be weak in our experiment as the signal is so small, and it will not cause any significant effect of photonics device performance.

Figure 7i–l show Ba 3d, Ti 2p, O 1 s and Ar 2p spectra obtained for BTO. In Fig. 7i, Ba 3d_5/2_ of the as-deposited sample (bottom red line) can be fitted to two peaks at BEs of 778.7 and 780.0 eV with ratio of 47%: 53%. After etching, Ba 3d_5/2_ is fitted to two peaks at BEs of 778.8 and 780.1 eV with ratio of 80%: 20%. No BE shift was observed after consideration of experiment error (± 0.2 eV). However, the surface oxide was removed due to etching process from peak ratio change. For Ti 2p, the BEs of Ti 2p_3/2_ before and after etching process are 458.1 and 458.2 eV, respectively, which is also within experimental error. For O 1 s spectra, before etching it can be fitted using two peaks with BEs of 529.4 and 531.4 eV, assigned to BTO and surface contamination, with ratio of 45%: 55%. After etching the BEs of peaks are 529.6 and 531.5 eV with ratio of 60%:40%, which shows that the surface contamination was removed. No obvious XPS peak from Ar is observed after etching in BTO, which accords well with the Z-cut and X-cut LN cases.

### Optical performance characterization and discussion

**Fig. 8:**
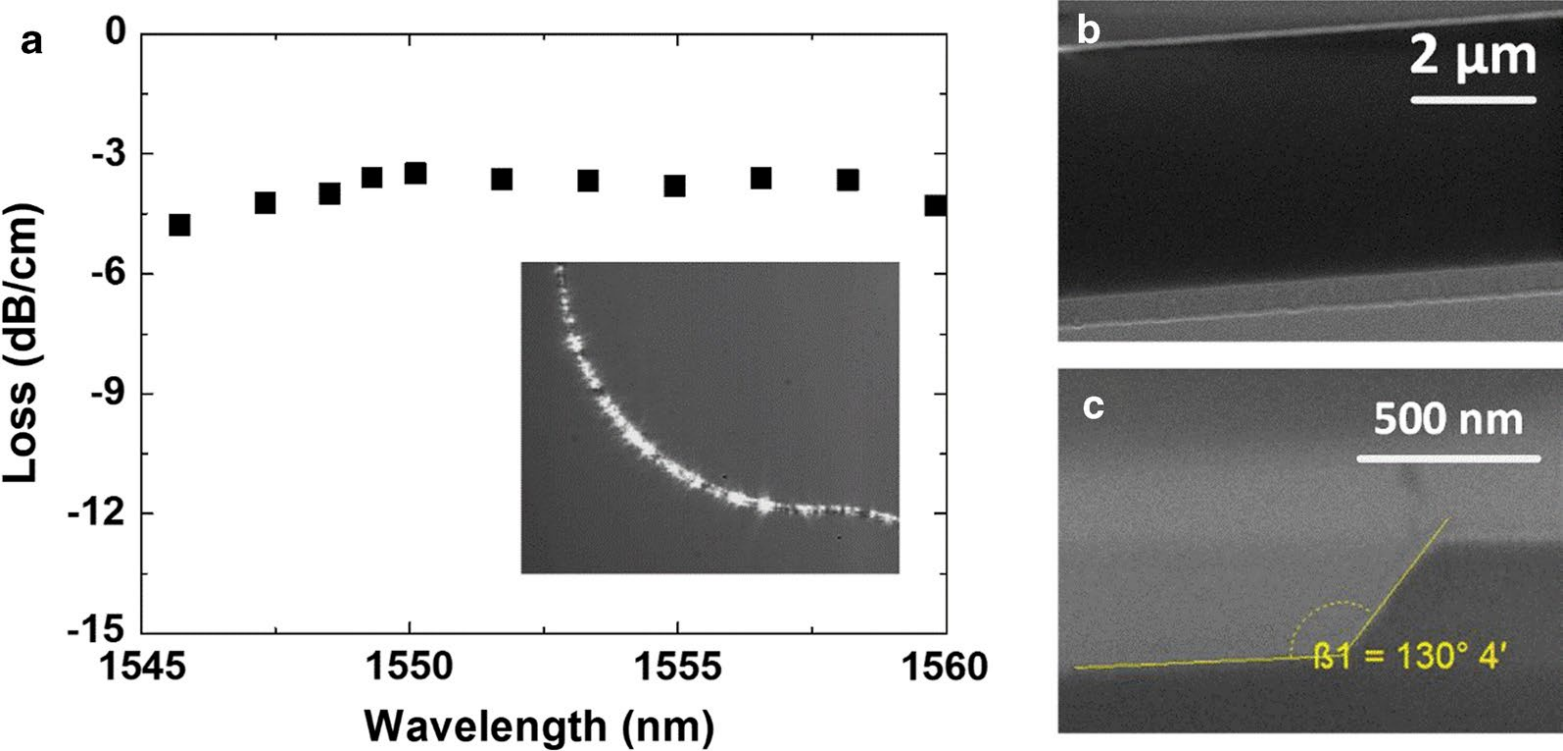
**a** Measured loss of the etched Z-cut LN waveguide. (The inserted picture shows the light transmitted inside the bend waveguide during measurement.) **b** SEM picture of the etched waveguide. **c** FIB picture of the waveguide cross section

Based on the optimized argon-based ICP method, Z-cut LN waveguides are realized as an example application. The waveguide is partially etched with an etch depth of 420 nm out of 700 nm total thickness, and its width is designed at 4 µm for easy lithography. The geometric dimensions of the waveguide are determined based on the 3D finite difference time domain (FDTD) method [17] after considering both the transmission loss and process technology. The top LN layer and substrate are isolated by 2 µm silicon oxide to form a high refractive index difference (the refractive indices of LN and SiO_2_ are, respectively, 2.3 and 1.44) for optical confinement, as the epitaxy shown in Fig. 1b. About -3.7 dB/cm propagation loss is measured with deducting of coupling loss (cutback method) near the design wavelength of 1550 nm for transverse magnetic (TM) polarized input light, as shown in Fig. 8a. The inset shows light confined well inside the waveguide. It's worth noting that there are other methods to characterize the waveguide loss, such as sliding-prism, Fabry–Perot resonances and scattered light methods [18]. The cutback method is used here. Figure 8b shows a scanning electron microscopy (SEM) image of the etched waveguide. Clear sidewalls validate the high-performance etching result. The sidewall angle is about 50 degree, as the focus ion beam (FIB) picture shown in Fig. 8c. The measured loss is reasonable for TM polarization (where the field is correctly aligned for maximizing the Pockels effect for the Z-cut orientation); and greater than typical loss values for transverse electric (TE) polarized light in X-cut lithium niobate [3, 6] due to the usual anisotropy in sidewall roughness. Such a loss can be improved by using thinner top LN thickness [19] and a more optimized structure [3, 6, 20]. It's worth noting that there is no post process or added cladding of the measured waveguide. The loss can thus be reduced by optimizing such processing, as is the case with addition of thermal oxidation in silicon waveguides [20, 21], or with use of gas cluster ion beam smoothening [22]. In the BTO case reduced index contrast between the DSO substrate and the top BTO layer (the refractive index of BTO and DSO are, respectively, 2.38 and 2.13, as determined by the prism coupling method) would result in poorer light confinement even though the etch is deeper; the loss cannot be directly compared to that in LN.

Compared with the typical ion diffusion method [12] for perovskite oxides in photonics applications, the argon-based ICP demonstrated in this manuscript can realize compact and high-performance integrated devices. As there are no etching reaction products in this method, the optical performance of the perovskite oxide crystal is not affected at all. Therefore, it's likely superior compared with fluorine or chloride-based ICP etching [13–15], which have been demonstrated for other kinds of applications, such as field effect transistors. ICP machines are widely used in industry, thus the yield of the proposed method will be high if every process step is controlled within a small margin of error.

## Conclusions

In conclusion, argon-based ICP etching for perovskite oxides is demonstrated in detail in this manuscript. The etching rates and its relationships to chamber pressure, gas flow, bias and ICP power are analyzed in Z-cut LN, X-cut LN and BTO. The measured results are regular and predictable, which will be useful for benchmarking all perovskite-based oxide etching, especially for photonics applications. The measured AFM results show that the surface roughness is better after argon-based ICP etching than before. Both EDS and XPS results show that such an etching method is a pure physical process and no etching residue is found on the etched surface. Some reasonable small peak shifts after etching are observed, but no significant performance degradation of the photonics devices is obtained during the experiment. A measured -3.7 dB/cm TM loss near 1550 nm for a Z-cut LN waveguide also validates the suitability of argon-based ICP for perovskite oxides etching in photonics device fabrication.

## Data Availability

All the data are fully available without restrictions.

## Abbreviations

ICP: Inductively coupled plasmas
LN: Lithium niobate
BTO: Barium titanium oxide
AFM: Atomic force microscopy
EDS: Energy-dispersive X-ray spectroscopy
XPS: X-Ray Photoelectron Spectroscopy
Cr: Chrome
e-beam: Electron beam
DSO: Dysprosium scandate
PLD: Pulsed laser deposition
RMS: Root-mean-square
BE: Binding energy
Pt: Platinum
Li: Lithium
Nb: Niobate
O: Oxygen
C: Carbon
Ar: Argon
Ba: Barium
Ti: Titanium
SEM: Scanning electron microscopy
FIB: Focus ion beam
TM: Transverse magnetic
TE: Transverse electric
